# Does Food Safety Risk Perception Affect the Public’s Trust in Their Government? An Empirical Study on a National Survey in China

**DOI:** 10.3390/ijerph16111874

**Published:** 2019-05-28

**Authors:** Guanghua Han, Simin Yan

**Affiliations:** School of International and Public Affairs, Shanghai Jiao Tong University, Shanghai 200030, China; ann1312911@163.com

**Keywords:** food safety, risk perception, governmental trust

## Abstract

This paper studies the impacts of food safety risk perception on the different dimensions of governmental trust. A logistic regression model was constructed based on the multidimensional analysis of government trust (i.e., competence, benevolence and honesty) with food safety risk perception, economic growth, combating corruption, social trust, political participation and demographic characteristics as explanatory variables. The main findings are that respondents with low levels of food risk perception, high political participation and a positive evaluation of economic growth and anti-corruption performance show high levels of trust in government competence, benevolence and honesty. Social trust has a spillover effect, which has a significant impact on government competence and benevolence but has no significant impact on the honesty of the government, which reflects the distinction between different dimensions of the public’s trust in their government. Highly educated people have low levels of trust in government competence, high levels of trust in government benevolence, and no significant impact on the judgment of government honesty. In general, the public speak lowly of the status of food safety and have limited interest in political participation. The government is better to strengthen food safety supervision and develop social capital to further enhance the public’s governmental trust.

## 1. Introduction

Currently, China is in a period of rapid economic growth, but some food producers’ non-standardization operations and simplistic pursuit of high revenue have caused some food safety incidents. The occurrence of food safety incidents in various countries has caused great damage to people’s physical and mental health. According to statistics by press, from 2004 to 2012, China’s cumulative exposure to food safety incidents totaled 2489, of which 16.5% involved multiple regions [1]. Frequent exposure to food safety incidents has evoked the suspicion of the regulatory capacity of local governments and sometimes seriously damaged governmental trust.

Food safety has a significant influence on governmental trust. With high risk and information-asymmetry attributes, foods are not able to be judged by consumers for either authenticity or quality [2]; therefore, the supervision of food safety by the government is required. In the food safety risk management process, consumers expect governments to provide information, especially bad news, about food safety rather than display a lack of positive action [3]. As an important part of the government’s regulatory responsibilities, food safety and reliability are important manifestations of the management capabilities of the government. In the past, when food safety incidents occurred, people generally attribute to government’s insufficient supervision and neglect of duty. Some scholars have pointed out that people’s concerns about food safety might seriously erode central and local governmental trust [4]. Governmental trust is a concept of psychological expectation. The essentials are the attitude, evaluation or belief and confidence of citizens in the government, the political system and government officials [5]. Governmental trust has a diffusion effect, and people’s lack of trust will lead to dissatisfaction with the government and a low efficiency of policy implementation [6], exacerbating social conflicts and causing social instability [7]. Therefore, it is necessary to further study the relationship between food safety risk perception and governmental trust.

In the existing literature, on the one hand, analysis of the relationship between food safety risk and governmental trust is mostly based on a single perspective of a Genetically Modified (GM) food survey [8,9,10,11], and there is relatively little research on the relationship between overall food safety risk and governmental trust. On the other hand, the discussion of governmental trust is too broad and lacks in-depth discussion of multiple dimensions of governmental trust, meaning people’s trust in the competence, benevolence, and honesty of the government [3,10,11,12,13]. Therefore, this paper attempts to extend food risk to the overall food safety issue and divides governmental trust into multiple dimensions for in-depth investigation, focusing on the multidimensional impact of overall food safety risks on governmental trust, providing more comprehensive and in-depth research results. This study will explore related research issues based on the theories of risk perception and governmental trust.

## 2. Previous Literature

Currently, China has the characteristics of a “risk society” [14], and social uncertainty is increasing. Risk has subjective constructiveness [15]. The concept of risk perception was first proposed by Bauer (1960) [16]. He pointed out that consumers are unable to determine the quality of the product at the time of purchase and actually bear a certain risk. It is emphasized that consumers’ behavior is driven by consumers’ subjective perception of the risk rather than the risk itself. Slovic (1987) argued that the concept of risk can be quantified [17]. They measured the influencing factors of people’s risk perception in multiple dimensions, including the controllability of risk, the severity of the consequences, the attributes of risk delay, and the knowledge of risk. In the field of food safety, there is a bias between the risk perception of the public and the actual risk level [18]. Overestimating and underestimating the actual risk exists simultaneously. The social amplification effect of risk also magnifies an accident to create an unknown risk and a potential threat, resulting in a direct impact beyond the disaster itself [17]. When a food safety incident occurs, the public generally believes that the government has a regulatory responsibility, and negative evaluations of food safety conditions will undermine public confidence in government regulatory capacity, leading to a crisis of governmental trust [4].

Trust is a topic of common interest in different disciplines such as public administration, sociology, psychology, economics, and political science [18]. The concept of governmental trust has also been widely discussed by scholars. In general, governmental trust refers to society members’ basic evaluation of government, political system and government personnel based on their own expectations [5,6]. Governmental trust is an interactive relationship between the public and the government. If the public is satisfied with the policy formulation of the government elite, then governmental trust will arise [19]. Governmental trust reflects the attitude of the public towards the government.

Governmental trust owns multiple connotations. For example, Norris (1999) argued that governmental trust is a multidimensional concept that can refer to the attitude of the public toward the state, the political system, and government agencies or government officials [20]. Hetherington (1998) incorporated government capacity into the category of government trust [6]; Levi (2000) believed that the government’s benevolence and predictability of government behavior are important components of government trust [21]; several scholars believed that integrity is closely related to government trust. Poortinga (2003) divided the dimension of governmental trust into two aspects [22]. One is the general trust factor, including ability, care, fairness and openness. The second is the doubt factor, reflecting the criticality attitude of the public on how government risk policies are formulated and implemented. Based on existing research, Grimmelikhuijsen (2012) divides the dimension of government trust into three aspects, competence, benevolence, and honesty, which refer to the public’s belief as to whether the government has the essential knowledge and skills for management, whether the government cares for the welfare of the people and whether the government keep their promises, respectively [23].

Because of the rich connotation of governmental trust, many studies have considered that the factors affecting governmental trust are numerous and complex. On the one hand, researchers in the perspective of institutions and performance believe that the governmental trust of the people is the result of judgment based on the comparison of material interests and is based on the expected utility perceived by the performance of government institutions [24]. People’s trust in the government is determined by the government’s ability to provide public goods. Government performance determines the degree of governmental trust which is the premise of governmental trust [25]. For example, macroeconomic levels, presidential attitudes, and changes in relevant decision-making in Congress can cause undulation of public trust in government [19]. In other words, the supporters of performance theory believe that the effectiveness of policy implementation, social stability, and the level of social and economic development cast great impacts on governmental trust. On the other hand, the influence of cultural factors and social capital have also been studied. Cultural interpretation believes that government trust is an extension of interpersonal trust, which stems from the different cultural values and social norms that individuals have shaped in their early socialization [25]. For example, there is a close relationship between social capital and well-established government trust, and public participation in public affairs is particularly important for government trust. Social capital is defined as the characteristics of social organizations, such as trust, norms, and networks. These factors enhance social efficiency in the form of joint action, and trust is a core element of social capital [26]. Keele (2007) believes that government trust is not the result of how the public views government and government officials, but how much people can participate in the performance of civic life, and is the result of interpersonal trust and reciprocal attitudes generated by citizens in their civic life [9]. In addition, the political and social environment, institutional arrangements, and public satisfaction, including demographic characteristics such as gender, age, economic income, and educational background, are all important factors influencing governmental trust. In general, in spite of huge differences in political and social cultures between countries, the factors affecting governmental trust can be classified into demographic characteristics, politics, government, economy, society, and culture [27].

The relationship between risk and trust is complex. There have been many empirical studies showing that the public’s risk perception and risk attitude determine their trust in the food itself and related institutions such as government and enterprises. Frewer (2003) believes that the risk information of food coming from industry associations, consumer organizations or government agencies is different, but the difference of information supply institutions has little effect on people’s attitude towards GM foods [10]. Consumers’ risk attitudes act on their level of trust in food. People’s attitudes toward GM foods determine the degree of trust, while non-institutional trust affects risk attitudes or risk perceptions. The study by Poortinga (2005) clarifies the decisive impact of risk judgment on trust [28]. He compared two perspectives of risk and trust relationship, the causal model and the associationism model of trust, and investigated whether trust is the cause of acceptability of GM foods (causal model) or outcome (association model). The results show that trust is the expression or predictor of the acceptability of GM foods. The general assessment based on risk perception and interest perception determines people’s trust level and supports the perspective of the association model. In addition, some empirical studies in recent years have also verified the direction of risk versus trust from other perspectives. For example, Meijnders (2009) found that the consistency of attitudes toward GM foods determines the degree of the public’s trust in journalists [11]; Liu (2014)’s research shows that scope of consumers’ trust objects are related to their concern level about food safety [12]. Consumers with low concerns trust doctors, personal experiences and research institutions, while moderate worriers only trust doctors and their own experiences [13]. It is also believed that food safety crises and public psychological risks have an impact on trust.

In the specific issue of food safety in China, there are also many studies that reflect the direct impact of people’s perception of food safety risk on governmental trust. As Yan (2012) pointed out, food safety issues present a spillover effect, involving many social, political and ethical issues outside the food sector [29]. Wu (2017) believes that with the improvement of China’s economic development level, people have a higher pursuit of quality of life and food safety [4]. The occurrence of food safety incidents has caused people’s dissatisfaction with government supervision and dereliction of duty, and impaired public confidence in supervision of the government, leading to the consequences of governmental trust crisis. These views intuitively illustrate the important influence of people’s perception of food risk on governmental trust.

After the combing of the existing literature, it can be found that on the one hand, the analysis of the relationship between food safety risk and trust is mostly based on the public’s perspective on the attitude of genetically modified foods, and the systematic research on the relationship between food safety risk and governmental trust is relatively rare [9,10,11,12]. The former study perspectives are relatively narrow. With the development of technology and industrialization, food safety risks are characterized by diversification. For example, hormone risks, additive risks, production and processing risks, etc., are intertwined. Therefore, it is necessary to proceed from the overall food safety situation and explore the impact of food safety risks on governmental trust. On the other hand, the research on the impact of food safety incidents upon public governmental trust is too broad. The existing research mainly discusses this from the perspective of broad governmental trust and lacks in-depth discussion on the multiple dimensions of governmental trust [9,10,11,12,13]. Governmental trust has rich connotations, such as competence, benevolence and honesty. It contains many aspects of people’s trust in government regulators [20,21,22,23]. The impact of food safety risks on the dimensions of governmental trust may also be different. Existing research cannot reflect the difference. Therefore, this paper will examine the impact of food safety risk perception on the three dimensions of governmental trust (competence, benevolence, and honesty) while incorporating economic growth, combating corruption, social trust, political participation, and demographic characteristics into explanatory variables to make an attempt to provide a comprehensive study.

## 3. Food Safety and Governmental Trust 

Governmental trust is often measured by three indicators, which include competence, benevolence, and honesty [23]. Specifically, governmental trust level refers to the public’s degree of trust in the effectiveness of the political system, the government’s concern for the welfare of the people, and the government’s honesty, respectively. Competence, benevolence, and honesty represent different levels of governmental trust. Competence [6] is a performance index of governmental trust, representing the public’s evaluation of the effectiveness of the political system and public policies as well as the administrative capacity of government officials. Benevolence [21] is an ethical indicator that represents the government’s concern for people’s livelihood and the interests of the people. It measures the motivations and goals of government management behavior. Honesty [23] is a decisive factor regarding the trust and credibility of the government. Once the people lose their trust in the government, there will be direct and serious damage to the credibility of the government and the governmental trust of the people. The three indicators together constitute an important aspect of measuring the different dimensions of governmental trust, which is of great significance. Therefore, this paper also chooses competence, benevolence, and honesty as explanatory variables to measure the levels of the people’s governmental trust.

As mentioned above, government performance and policy implementation have the most important effect on governmental trust. The level of food safety supervision reflects the government’s public management ability. The lower the level of public food safety risk perception is, the higher the satisfaction will be given with the food safety status. The higher the public’s evaluation of the government’s ability to perform public management duties is, the higher their levels of governmental trust. Second, food safety is one of the most relevant topics in the daily lives of residents and is closely related to public health and quality of life [4]. The emphasis on food safety issues reflects the government’s concern for public health and well-being. Therefore, the higher the quality of life is, the higher the trust in the government’s benevolence. At the same time, the asymmetry of food information gives the government the responsibility of food safety information disclosure and risk communication. After a food safety incident, the transparency of risk information directly affects the people’s risk perception levels, and the government’s choice to conceal the security incident information or not reflects the level of the government’s honesty. Therefore, based on the effectiveness of the administrative system, the degree of government care for public welfare and whether the government is open and honest, the public’s perception of food safety risk most probably has an impact on their level of governmental trust. These hypotheses are given:

**H1a:**  All else being equal, people with a lower perception of food safety risks are expected to show higher levels of trust in government competence;

**H1b:**  All else being equal, people with a lower perception of food safety risks are expected to show higher levels of trust in government benevolence;

**H1c:**  All else being equal, people with a lower perception of food safety risks are expected to show higher levels of trust in government honesty.

The proponents of “performance theory” also believe that successful government intervention in economic market failures can help increase governmental trust, while failed interventions reduce governmental trust [19,24,25]. The government’s positive evaluation of economic growth, the welfare of people’s livelihood, and governance performance in the field of pure public goods all contribute to governmental trust. The economic development status and economic performance determine the public’s evaluation of the government’s macroeconomic regulation and control ability. A higher evaluation of the economic development status reflects the government’s strong ability to allocate resources and macroeconomic regulation and people’s great trust in their political system and government. The better the economic development is, the higher the income and living standards of the people, and greater recognition of government performance and government care will be given. At the same time, a good economic market order is inseparable from open and transparent market information. The government’s supervision of open market information and the active disclosure of bad information can help to increase public governmental trust. The more the economic market operates in a transparent and orderly manner, the higher the level of trust in honesty will be. Therefore, we put forward the hypotheses:

**H2a:**  All else being equal, people with greater perceptions of economic development are expected to show higher levels of trust in government competence;

**H2b:**  All else being equal, people with greater perceptions of economic development are expected to show higher levels of trust in government benevolence;

**H2c:**  All else being equal, people with greater perceptions of economic development are expected to show higher levels of trust in government honesty.

In addition, political performance factors affecting governmental trust include the government’s crackdown on corruption. Corruption has a significant impact on governmental trust. The higher the degree of corruption, the more negative the public’s performance evaluation of the political system. The prevalence of corruption will directly weaken the level of governmental trust, and anti-corruption will contribute to the improvement of governmental trust [30]. Political corruption directly challenges and undermines the rules of political institutions, affects the standardized operation of the market, and poses a serious threat to economic development and political performance. The greater the government’s crackdown on political corruption is, the higher the public’s trust in the political system and government capacity. At the same time, the taxation of taxpayers’ taxes by corrupt officials will directly harm people’s public welfare and well-being, and this will easily lead to public dissatisfaction, resentment and even group incidents. The greater the government’s crackdown on corruption is, the more it will help to safeguard the rights and well-being of the public, and the higher the public’s trust in the government’s reputation will be. At the same time, corruption is a black-box operation, and it is a very destructive act against political cleanliness and openness. The outbreak of political corruption scandals will greatly affect the public’s evaluation of the honesty of government officials [31], resulting in a serious decline in public trust in the government. Therefore, we put forward these hypotheses:

**H3a:**  All else being equal, people with a higher evaluation of a crackdown on corruption are expected to show higher levels of trust in government competence;

**H3b:**  All else being equal, people with a higher evaluation of a crackdown on corruption are expected to show higher levels of trust in government benevolence;

**H3c:**  All else being equal, people with a higher evaluation of a crackdown on corruption are expected to show higher levels of trust in government honesty.

The proponents of “social capital theory” believe that social capital has a significant impact on governmental trust. Governmental trust and social trust are closely linked. The influence of social capital mainly comes from the social network established by the autonomous organization and the reciprocal norms and trust between citizens [27], and people who trust others more often show greater governmental trust. Governmental trust is partly the result of social trust. In other words, people’s sense of trust has commonality and interactivity, and social trust and governmental trust influence each other.

**H4a:**  People who trust others and society more are expected to show higher levels of trust in government competence;

**H4b:**  People who trust others and society more are expected to show higher levels of trust in the government’s benevolence;

**H4c:**  People who trust others and society more are expected to show higher levels of trust in government honesty.

In addition, the factors that influence governmental trust include political participation. Some scholars have pointed out that public participation affects governmental trust through five intermediaries including consensus building, ethical behavior, responsible practice, service ability and management ability [32]. Good political interactions help to strengthen communication and collaboration between the government and the public, thereby enhancing government trust. For example, village elections, village self-government, and opinions expressed by urban residents in China. Public participation can raise public awareness of political events and public affairs, raise public comprehension of policy development and policy implementation, enable people to better understand policies and implementation intentions, and enhance trust in government competence and government benevolence. In the process of public participation, the openness and transparency of the rules during hearing and voting help the public to feel the fairness and sense of participation. The more positive feedback on political participation is, the greater the enthusiasm of the people for political participation, thus forming good political participation in the circular mechanism. The enthusiasm of public political participation also reflects its evaluation of the credibility of the government in democratic voting. The higher the enthusiasm of participation, the more it reflects trust in the government’s honesty, so the following hypotheses are put forward:

**H5a:**  People who have higher levels of public participation are expected to show more trust in government competence;

**H5b:**  People who have higher levels of public participation are expected to show more trust in government’s benevolence;

**H5c:**  People who have higher levels of public participation are expected to show more trust in government honesty.

In addition to the above influencing factors, some studies examined the extent to which demographic characteristics act as control variables for governmental trust. Based on empirical research, Li (2004) found that gender, education, age and family income have little significant impact on government trust [33]. An empirical study based on the 2013 “China Urban Residents Values” survey data, shows that gender and income have no significant impact on governmental trust, while age and education have a negative impact on governmental trust. Among the variables, the degree of education has always been the focus of scholars’ study on governmental trust. Highly educated people tend to establish government trust through a rational evaluation of politics, while less educated people build government trust through general non-differentiated social attitudes [34]. In general, most studies take demographic data into consideration for the model, but there are significant differences in the evaluation of the impact of provincial factors. In this study, variables such as gender, age, marital status and education level are included as control variables to verify whether they have an impact on governmental trust. 

## 4. Empirical Analysis

### 4.1. Data and Methods

Data employed in this study is collected by the Asia Barometer Project in its fourth round of Asian Barometer Surveys (4th ABSs). The survey with a questionnaire consisting of over 200 questions was conducted through face-to-face interviews from July 2015 to March 2016. In the survey, a total of 125 primary sampling units (PSUs) were used based on the Census Yearbook from the China National Statistics Bureau, and respondents were randomly selected from PSUs with a representative sampling method of probability proportional to size. The target population covers Chinese people aged 18 and above who have the ability to respond and have resided in the surveyed area for at least one month. Specifically, people who were residing in certain places were not considered in the survey, i.e., military residential complexes, residential units in compounds of central ministries, embassies and consulates, infrastructural buildings such as power stations, wind stations, prisons, tourist destinations and religious sites. A total of 6013 eligible samples were taken in the field. After interviews were completed, at least three rounds of validity checks were successively undertaken on every questionnaire by the interviewer, the supervisor and the data manager in ABSs Centre office. The standard for the validity check includes the correctness of reaching the target interviewee and standardization in the interviewing process, the ability of the interviewee to understand and answer the questionnaire, and the reliability of the interviewee’s response. To conduct this study, we excluded the questionnaires with uncompleted questions regarding our variables, and 1273 completed and valid interviews were obtained in mainland China.

### 4.2. Descriptive Statistics

The independent variables include the public’s evaluation of food safety risks, economic development, the government’s anti-corruption performance, people’s social trust, political participation, and demographic characteristics. The dependent variable includes three dimensions of governmental trust: competence, benevolence, and honesty of the government. Because most questions are 4-classification responses which aim to measure the attitude or evaluation of the interviewee, the difference between categories is not obvious, and the distinct degree of judgment on the public attitude and the trust of the government is not intuitive, so “strongly agree” and "agree" or “always vote” and “usually vote” are classified into positive categories and “strongly disagree” and “disagree” or “sometimes vote” and “never vote” are classified into negative categories. Thus, the 4-class variables are transformed into dichotomous variables, making the judgment of the interviewee as having a positive or negative attitude more intuitive. After the conversion, the definition of each variable and descriptive statistics are shown in Table 1 (see the Appendix A for the problem setting of the variables).

From the descriptive statistics, it can be seen that compared with economic growth, respondents have relatively lower evaluations of China’s food safety status (average score is only 5.09), and the public’s perception of food risk is relatively high. The evaluation of China’s economic growth is relatively higher (average 6.72), reflecting the public’s worries about the status quo of food safety and the recognition of the government’s macroeconomic regulation and control capabilities. Ninety-two percent of the respondents believe that the Chinese government has made every effort to crack down on political corruption in the past three years; only 8% of the people said that the government had not fulfilled its duty to fight corruption. In general, most people recognize the government’s efforts to combat political corruption and believe in its determination and perseverance in recent years. Eighty-three percent of respondents believe that most people are trustworthy, and 17% oppose this view, which indicates that most people have higher levels of social trust and interpersonal trust, but there are still some people who have lower levels of social trust. The overall level of social trust is high. It is noteworthy that only 50% of the respondents indicated that they frequently participated in the democratic elections. Half of the respondents indicated that they were less involved in the democratic vote or have never voted, which reflects people’s lack of interest in the community elections. The phenomenon of low intention of political participation is very obvious in China. In terms of demographic characteristics, 42% of the subjects are female and 58% are male, and the gender distribution is relatively balanced. In terms of age, there is a certain number of respondents in all age groups, and the number of middle-aged and older respondents is relatively large (average age 43.7 years). The age distribution is also reflected in the finding that most of the respondents have cohabiting experience (approximately 81%). Overall, the respondents had a low level of education, and the average length of education was approximately 10 years.

Respondents’ overall trust in China’s political system and government capacity is high, with 82% of respondents believing that China’s current political system can solve China’s main problems, which reflects their confidence in and approval of national strength, the political system and government capacity. However, 31% of the respondents believe that the government has not responded to the immediate needs of the people and has provided insufficient care for the interests and well-being of the people. This reflects the fact that despite the high level of trust in the political system, the levels of satisfaction and trust in government benevolence is low. Additionally, it is noteworthy that 45% of the respondents believe that the government sometimes conceals important information from the public, and the public’s trust in the government’s transparency and honesty is not sufficient. This results reflect public’s worry that the local governments are often concerned with maintaining social stability and steadiness, and they have intention to cover up the truth and blocking negative news. This reflects that the government’s work on information disclosure needs to be further strengthened to enhance people’s trust in government integrity and transparency. It can be seen from the above results that there are significant differences in the various aspects of public trust in the government, and it is necessary to conduct multidimensional research on government trust.

## 5. Results

### 5.1. Government Competence

The regression results of the competence dimension of governmental trust are shown in Model 1 and Model 2. It can be seen that the regression results of Model 1 and Model 2 are both qualified. The Nagelkerke R-squares are all above 0.15, and the pseudo-R-square of Model 2 is 0.17, indicating that the overall fit of the model is relatively high-quality. In Model 2, which contains control variables of demographics, the regression coefficient of food safety status assessment is 0.137, which indicates that people with a lower food-risk perception have a higher level of trust in government competence. This means the hypothesis H1a was validated, and the regression results were significant (*p* < 0.01). In terms of economic growth, the regression coefficient of economic development evaluation is 0.226, which reflects that respondents with better evaluation of economic development have higher trust in government capacity. The research hypothesis H2a is verified, and the regression results are significant (*p* < 0.01). This validates the views that the improvement of government performance and material interests will help promote governmental trust. Combating corruption (regression coefficient of 0.502) has a significant positive effect on people’s trust in government competence. Hypothesis H3a is verified, and cracking down on corruption can help people’s recognition of government capacity. Social trust (regression coefficient 0.557) has a significant impact on government competence, and hypothesis H4a is validated. The higher the respondent’s trust in others is, the higher the degree of trust in government capacity, and to some extent, that social trust has spillover effects. The political participation regression results (regression coefficient 0.422) also verified the research hypothesis H5a. The more active the voting is in democratic elections, the higher the trust in government competence will be. In general, there is a significant correlation between the selected independent variables and the public trust of government competence, and the model regression results are significant.

### 5.2. Government Benevolence

Model 3 and Model 4 reflect the regression results of the benevolence dimension of governmental trust. The Nagelkerke R square is approximately 0.10, and the regression results of each variable have a significance level of 0.05 or higher, which indicates a significant correlation. In Model 4, the food safety coefficient is 0.140, which indicates that the food safety status evaluation has a positive correlation with the government’s benevolent trust. The higher the food safety perception is, the more people trust the government’s reputation. Economic development (regression coefficient is 0.091) and anti-corruption (regression coefficient is 0.861) have positive impacts on improving trust in the government’s benevolence. The government’s achievements in economic development and corruption will help increase public trust in the government’s benevolence. In addition, the impact of anti-corruption is relatively strong (i.e., the regression coefficient is greater). The two variables of social trust (regression coefficient, 0.359) and political participation (regression coefficient, 0.319) also have a positive effect on the benevolence dimension of government trust. People with high levels of social trust and active political participation are more likely to believe that the government cares about the welfare of the people.

### 5.3. Government Honesty

The regression results of governmental trust in the honesty dimension are reflected in Model 5 and Model 6. Compared with the competence and benevolence dimensions, the relationship between the honesty dimension of governmental trust and the independent variables shows a significant difference. In Model 6, food safety perception (regression coefficient, 0.137), economic development (regression coefficient, 0.105) and anti-corruption (regression coefficient, 1.063) have a significant positive effect on governmental trust in government honesty (significance level, 0.01). Relatively speaking, anti-corruption has the greatest impact. The regression coefficient of political participation is 0.245, which is positively related to the trust of the government’s integrity, but the significance level is only 0.1. Finally, it is worth noting that the influence of social trust on the evaluation of government honesty is not significant, which directly reflects that the impact of independent variables on different dimensions of governmental trust is distinctive.

**Figure 1:**
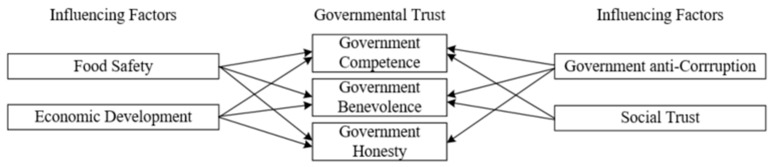
Governmental trust and its influencing factors.

The supported hypotheses in the empirical analysis are summarized in Table 2. Since governmental trust includes several dimensions, the influencing factors to each dimension of governmental trust are presented in Figure 1. 

## 6. Discussion 

Based on the research results obtained, this paper will propose corresponding policy recommendations. First, food safety risk research assumes that H1a, H1b and H1c are verified. This shows that in the opinion of the public, food safety supervision is an external manifestation of the government’s public management capacity. The better the food safety perception is, the stronger the government’s regulatory capacity will be, and the more people will trust the government’s ability to perform public management duties. In addition, the improvement of food safety status reflects the government’s response to the welfare and needs of people, as food safety is closely related to the health and safety of residents. Every time a large-scale food safety incident breaks out, social panic results, which leads to a decline in governmental trust. The crisis of social trust and the improvement of the food safety situation are conducive to enhancing trust in the government’s benevolence. The impact of food safety is also reflected in trust in the government’s honesty: the better the food safety and the more transparent food safety risk information are, the higher the level of people’s trust in the government’s honesty. At present, as the overall evaluation of the food safety status is low, further strengthening food safety supervision and improving food safety potentially have a significant positive effect on the promotion of public governmental trust. 

In terms of economic growth, respondents who have a better evaluation of economic development have higher trust in government competence, benevolence and honesty. This finding validates that government performance and economic interests promote governmental trust. Economic growth is not only about the increase and expansion of wealth but also reflects the growth of people’s actual welfare and the improvement of their quality of life [35]. Meanwhile, the sustainability of economic growth depends on the transformation and optimization of the economic structure, where the government plays an important role in controlling the overall economic trends, economic regulation and the optimization of resource allocation. As a result, good economic growth is an important manifestation of the government’s capacities. Therefore, people who have higher evaluations of economic growth have higher levels of trust in government competence, benevolence and honesty, which shows that economic growth has a comprehensive impact on the improvement of governmental trust. Paying attention to economic development is still an important way to improve government trust.

The political performance factors also include the government’s efforts to combat corruption. According to the regression results, anti-corruption has a significant positive effect on enhancing governmental trust, which is respectively reflected in government competence, benevolence and honesty. This shows that the government combating political corruption is of great significance. Corruption causes serious damage to the political system and social fairness, which results in great waste of social resources and great damage to the interests of the people, thereby seriously weakening the people’s levels of governmental trust. Combating corruption shows the government’s maintenance of fairness and justice as well as the management and supervision of government officials. It reflects the effectiveness of the political system [36], the protection of the public’s interests and the emphasis on officials’ honesty and integrity. Compared with economic growth and food safety, the coefficients of anti-corruption and governmental trust are even greater. This shows that anti-corruption has a more significant effect on enhancing governmental trust. The government should focus more on strengthening the construction of an honest government and improving the transparency of the administration.

The influence of social trust on governmental trust is special. Social trust has a significant impact on government capacity and benevolence but has no significant impact on government honesty. The establishment and development of social capital will help to improve the relationship between individuals and groups. In addition, more than half of the respondents are from rural areas, where local communities are rife with acquaintances, and social trust is higher than it is in cities. In recent years, the quality of life of farmers has been greatly improved due to the government’s preferential policies. Therefore, respondents with higher social trust tend to have a high degree of trust in the government’s competence and benevolence [37]. However, the improvement of social trust has no significant relationship with the judgment of government honesty. The government’s honesty only reflects whether governmental information is transparent and whether the government keeps its promises, while the public’s trust in society and others has no impact on the assessment of the government’s honesty. In addition, the results show that different dimensions of governmental trust have significant distinctions. 

The regression results of political participation and governmental trust also validate the research hypothesis. The more active and politically involved voters in democratic elections are, the higher their trust in government competence, benevolence and honesty. This reflects the fact that political participation helps facilitate communication between the public and the government. Citizens influence the political activities of interest through democratic voting, which reflects their recognition and trust in the current political system and the use of existing political rules as well as the need for policy to maximize their own interests. Therefore, the higher the degree of political participation is, the higher the level of trust in the effectiveness of the political system and the government’s response to public demands. At the same time, public participation can help people become more active in understanding policy goals and implementation intentions better and help them to obtain more policy information. At present, the Chinese government is actively carrying out a disclosure of governmental information, but many residents are unaware of how to obtain public information [38]. People who have high rates of political participation and active participation in democratic voting will receive more public policy information, so their levels of trust in government honesty are higher. Openness and transparency will encourage people to exercise their right to vote and increase the enthusiasm of political participation, thus enhancing governmental trust.

In addition to the influencing factors above, the regression results of demographic variables are also distinctive. Men are more convinced of the effectiveness of the political system and the ability of government, but gender has no effect on the benevolence and honesty dimensions of governmental trust. The older the respondents are, the higher their trust in government competence, benevolence and honesty. It is worth noting that the influence of the educational level that scholars focus on has specific impact on governmental trust. As mentioned earlier, some scholars believe that highly educated people tend to build government trust through rational evaluation of politics, and less educated people build government trust through general non-differentiated social attitudes [34]. According to the regression results, the higher the degree of education is, the lower their trust in the government’s competence but the higher the trust in the government’s benevolence, which agrees with the aforementioned scholars’ point of view to some extent. The judgment of government competence is based on rational evaluation. Highly educated people often look at the political system and officials with a critical attitude. Therefore, it is easy for highly educated people to form a negative assessment of government capacity [39,40]. Despite their lack of trust in government capability, highly educated people have a higher degree of trust in government benevolence, which reflects their recognition of the government’s initial goals in responding to people’s needs and government work. In addition, the regression between education and the level of trust in government honesty is not significant.

## 7. Conclusions

This study focuses on the influences of public’s risk perception of food safety to governmental trust. Three indicators include competence, benevolence and honesty are employed to measure governmental trust. After an empirical study, we find that public’s perception of food safety do have strong affects on governmental trust in every indicator, reducing the general public’s perception of food risk is a way to enhance governmental trust in practice of public administration. 

Since governmental trust is a complex and connotative concept, there are many factors influencing it. Investigating governmental trust offers many potential research opportunities. For example, government regulations might influent the public’s perception of governmental abilities, which sometimes directly links with governmental trust. Thus, a multiple level study on government regulations of food safety to governmental trust is a potential research direction in the future. In general, governmental trust is a multidimensional, multilevel concept. To enhance government trust, the government should carry out multifaceted work and continuous efforts.

## Figures and Tables

**Table 1:** Variable definition and descriptive statistics.

Variable	Definition	Measurement	Frequency/Average	Cumulative Percent
Independent variables	Food safety perception	1–10, 1—very bad, 10—very good	5.09 (mean)	/
	Economic development	1–10, 1—very bad, 10—very good	6.72 (mean)	/
	Government anti-corruption	0—good	1153	92%
		1—bad	120	8%
	Social trust	0—high	1052	83%
		1—low	221	17%
	Political participation	0—active	641	50%
		1—inactive	632	50%
	Gender	0—female	533	42%
		1—male	740	58%
	Age	Actual age	43.72 (mean)	/
	Cohabitation Experience	0 = Do not have	241	19%
		1 = Have	1032	81%
	Education	Educational years	9.9 (mean)	/
Dependent variables	Government competence	0—good	1043	82%
		1—bad	230	18%
	Government benevolent	0—good	873	69%
		1—bad	400	31%
	Government honesty	0—good	694	55%
		1—bad	579	45%

**Table 2:** Logistic regression results for each dimension of governmental trust.

Category of Variable	Variable	Competence B (Standard Error)		Benevolence B (Standard Error)		Honesty B (Standard Error)	
		Model 1	Model 2	Model 3	Model 4	Model 5	Model 6
**Explanatory Variable**	Food safety perception	0.145 *** ^1^ (0.034)	0.137 *** (0.035)	0.133 *** (0.027)	0.140 *** (0.028)	0.148 *** (0.028)	0.137 *** (0.026)
	Economic development	0.233 *** (0.043)	0.226 *** (0.044)	0.098 *** (0.035)	0.091 *** (0.035)	0.123 *** (0.033)	0.105 *** (0.034)
	Anti-corruption	0.491 ** (0.236)	0.502 ** (0.238)	0.886 *** (0.202)	0.861 *** (0.204)	1.062 *** (0.215)	1.063 *** (0.216)
	Social trust	0.598 *** (0.182)	0.557 *** (0.184)	0.377 ** (0.160)	0.359 ** (0.162)	0.244 (0.157)	0.206 (0.159)
	Political participation	0.538 *** (0.162)	0.422 ** (0.170)	0.321 ** (0.129)	0.319 ** (0.135)	0.355 *** (0.121)	0.245 * (0.126)
**Control Variable**	Gender		0.332 ** (0.161)		−0.005 (0.129)		0.069 (0.122)
	Age		0.014 ** (0.006)		0.008 * (0.005)		0.017 *** (0.004)
	Cohabitation Experience		−0.091 (0.199)		0.253 (0.174)		0.162 (0.163)
	Education		−0.017 ** (0.009)		0.028 ** (0.014)		−0.006 (0.008)
	Constant	−1.793 ***	−2.165 ***	−1.774 ***	−2.385 ***	−2.727 ***	−3.213 ***
	-2 Log likelihood	576.564	1061.618	689.835	1478.857	705.787	1608.409
	Nagelkerke R^2^	0.151	0.170	0.099	0.109	0.124	0.141

^1^ Significance level, * *p* < 0.1,** *p* < 0.05,*** *p* < 0.01.

## Appendix A

**Table A1:** Variables and Measures.

Variables	Definition	Questions	Measurement
Independent variables	Food safety perception	What do you think of the current food safety situation in China?	Score 1 to 10 Higher score means better perception of food safety
	Economic development	What do you think of China’s current economic growth?	Score 1 to 10 Higher score means better perception of economic growth
	Anti-corruption	Has the government made enough efforts to fight corruption in the past three years?	Yes, I agree
			No, I do not agree
	Social trust	In general, most people can be trusted?	0—agree
			1—disagree
	Political participation	Since you are qualified, have you voted in the election so far?	0—Yes, I have
			1—No, I have not
	Gender	(0—female, 1—male)	0—female
			1—male
	Age	Actual age	Actual age
	Cohabitation Experience	Do you have cohabitation experience (including married, living with unmarried lover)?	0 = I do not have
			1 = I have
	Education status	How many years you have been educated in schools?	Years of education
Dependent variables	Government competence	In the long run, can China’s political system solve the main problems?	0—agree
			1—disagree
	Government benevolence	The degree to which the Chinese government responds to the needs of the general public	0—response given
			1—no response
	Government honesty	Our government often hides important information from ordinary people?	0—disagree
			1—agree
